# Development of a droplet digital PCR method for detection of *Streptococcus agalactiae*

**DOI:** 10.1186/s12866-020-01857-w

**Published:** 2020-06-23

**Authors:** Yi-Fan Zeng, Chu-Mao Chen, Xiao-Yan Li, Jun-Jiang Chen, Yan-Ge Wang, Shi Ouyang, Tian-Xing Ji, Yong Xia, Xu-Guang Guo

**Affiliations:** 1 Department of Clinical Laboratory Medicine, The Third Affiliated Hospital of Guangzhou Medical University, Guangzhou, 510150 China; 2 Institution of Kingmed of Guangzhou Medical University, Guangzhou, 510000 China; 3 Key Laboratory for Major Obstetric Diseases of Guangdong Province, Guangzhou, 510150 China; 4 iHuman Institute, ShanghaiTech University, Shanghai, China; 5 The Fifth Affiliated Hospital, Southern Medical University, Guangzhou, 510900 China; 6 Department of Infectious Disease, The Fifth Affiliated Hospital of Guangzhou Medical University, Guangzhou, 510000 China; 7 Department of Clinical Medicine, The Second Affiliated Hospital of Guangzhou Medical University, Guangzhou, 511436 China; 8 Department of Clinical Medicine, The Third Clinical School of Guangzhou Medical University, Guangzhou, 511436 China; 9 Key Laboratory of Reproduction and Genetics of Guangdong Higher Education Institutes, The Third Affiliated Hospital of Guangzhou Medical University, Guangzhou, 510150 China; 10 Stomatological Hospital of Guangzhou Medical University, Guangzhou, 510150 China

**Keywords:** Droplet digital PCR, *Streptococcus agalactiae*, Quantitation

## Abstract

**Background:**

*Streptococcus agalactiae* (GBS) is the causative pathogen of puerperal sepsis in pregnant women and pneumonia, sepsis and meningitis in infants. Infection of GBS is responsible for the increased morbidity in pregnant women and the elderly, and bring challenges to clinical diagnosis and treatment. However, culture-based approaches to detect S.agalactiae is time-consuming with limited sensitivity. Besides, real-time quantitative PCR demands expensive instruments with tedious steps. Thus, we aim to establish a new detection method for more accurate and rapid detection of S.agalactiae.

**Results:**

The ddPCR primer targeted the *CpsE* gene showed better amplified efficiency in the reaction. The limit of detection for GBS DNA with ddPCR was able to reach 5 pg/μL. Moreover, no positive amplified signals could be detected in the reactions which served 11 non-GBS strains DNA as templates. Furthermore, the coefficient of variation of this method was 4.5%, indicating excellent repeatability of ddPCR assay.

**Conclusions:**

In our study, ddPCR was performed as a rapid detection of S.agalactiae with high sensitivity and specificity. This technique can promote the accuracy of the diagnosis of GBS infection and provide a scientific basis for clinical treatment.

## Background

*Streptococcus agalactiae* (Group B Streptococcus, GBS) is a facultative anaerobic gram-positive opportunistic pathogen, which colonizes the gastrointestinal and genitourinary tract of approximately 30% of the healthy adults [1]. Moreover, infection with GBS is the main cause of pneumonia, septicemia and meningitis in neonates, and is especially responsible for the high morbidity rate of pregnant women [2–4]. So far, detection of *Streptococcus agalactiae* varies from culture-based methods to novel molecular tools [5, 6]. Traditional culture is laborious and time-consuming with limited sensitivity. Although real-time qPCR and other rapid techniques, such as MALDI-TOF-MS, are now commercialized, the cost and expertise limit their use in most laboratories [7]. Therefore, early diagnosis of infection requires a novel method with rapid and specificity in the detection of GBS.

Recently, droplet digital PCR (ddPCR) has been utilized in quantifying nucleic acid and detecting pathogens [8–10]. This method dilutes and divides the mixtures into many microdroplets with oil. Each microdroplet is amplified as an independent reaction system with or without target genes. The amplified condition of ddPCR is similar to that of real-time PCR with the probe for signal detection. Eventually, the absolute concentration will be calculated precisely according to the Poisson distribution without a standard curve [11–14].

Droplet digital PCR is an ultraprecise, reliable and economical method in the diagnosis of infectious disease. However, detection of GBS based on ddPCR has not been reported yet. Thus, we aim to evaluate the ddPCR for the detection of GBS and test whether ddPCR can be an alternative assay for the rapid diagnosis of GBS infection.

## Results

### Primer screening test

**Fig. 1:**
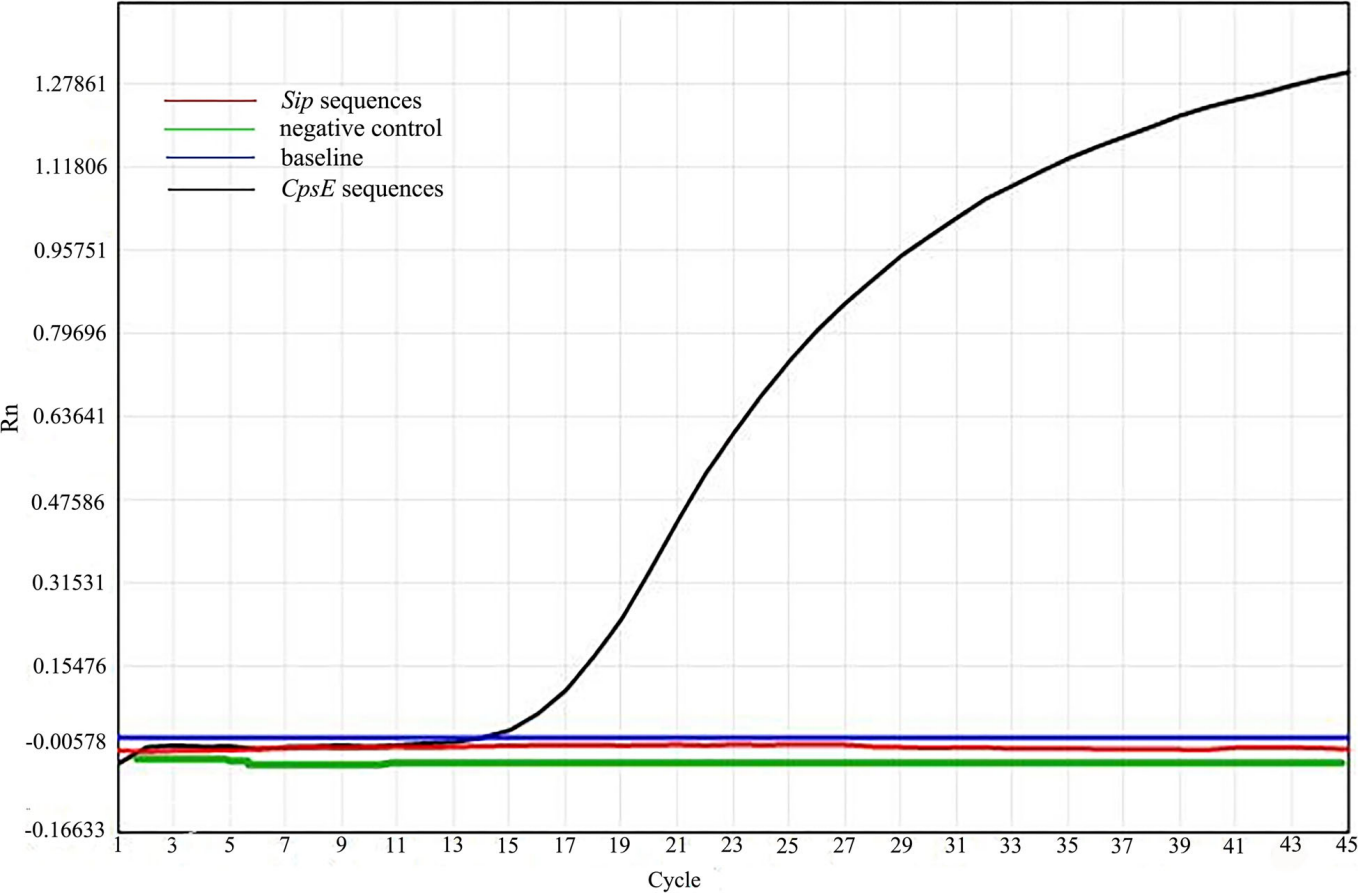
The primer of the qPCR screening experiment in the present study

Two sets of primers showed different amplification to GBS ATCC13813 monitored by the SLAN-96P real-time system (Fig. 1). The amplified signal was firstly detected 15 cycles after the reaction and the peak emerged at approximately 45 cycles with *CpsE* primer. No other amplification was seen in the *Sip* primer and negative control. Therefore, the *CpsE* primer was selected for the subsequent tests.

### Sensitivity and specificity of ddPCR for GBS

**Fig. 2:**
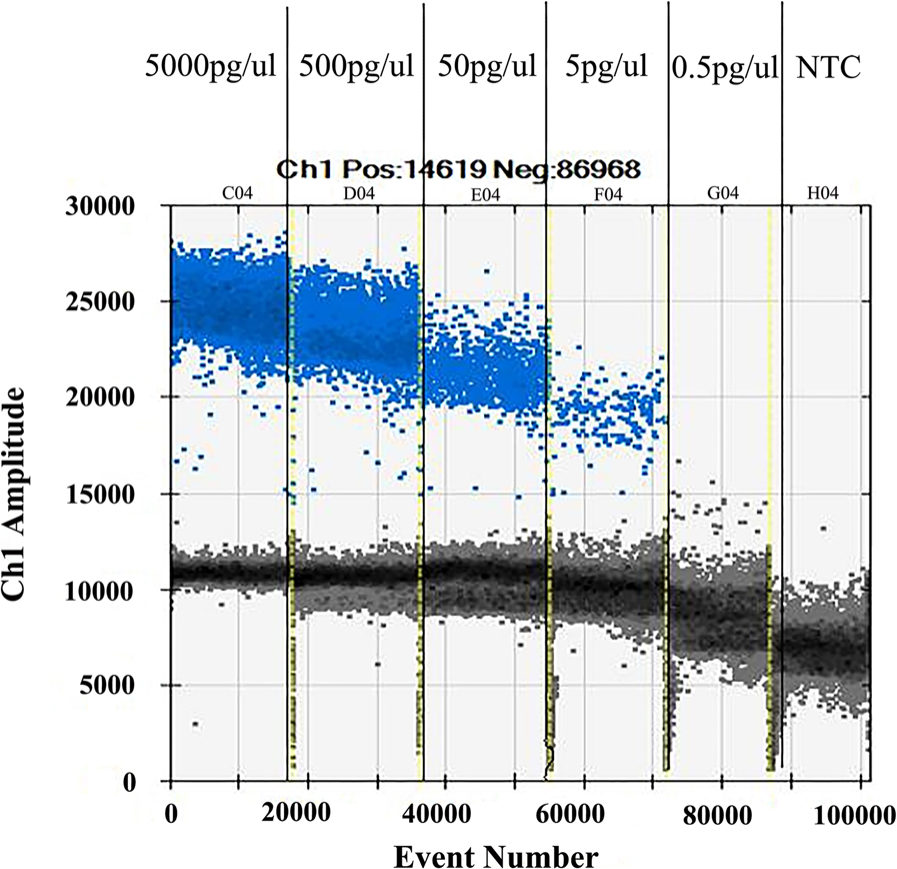
Sensitivity assay for ddPCR using continuous 10-fold dilutions of DNA templates from GBS ATCC13813

**Fig. 3:**
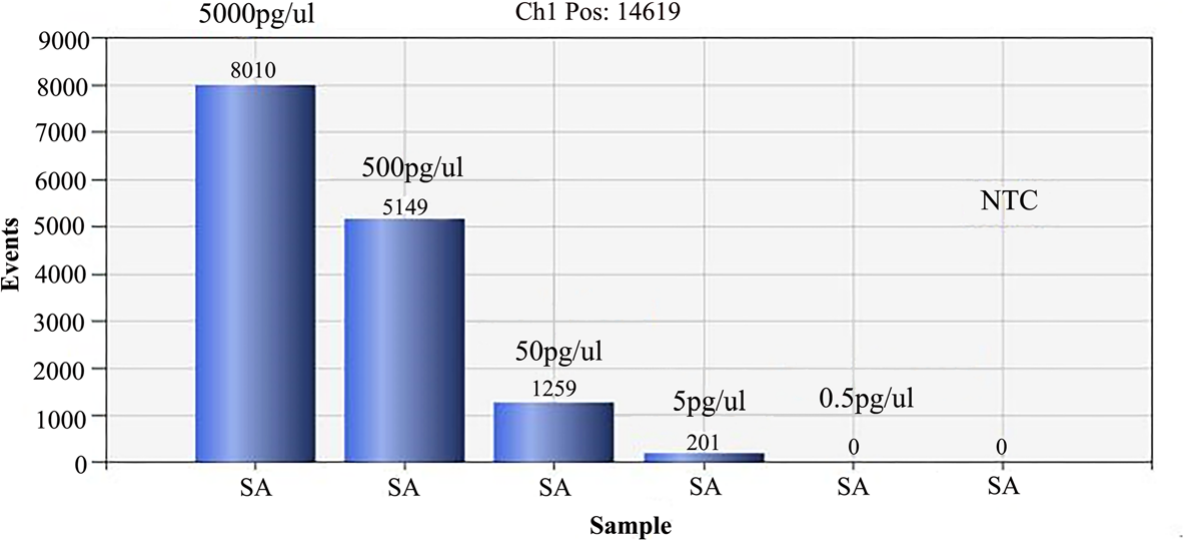
The number of events in each amplification concentration

The limit of ddPCR for detecting GBS DNA was able to reach 5 pg/μL. As shown in Fig. 2, the horizontal axis represented the event number of four concentrations templates, and the vertical represented the sample amplitude. The positive and negative microdroplets were shown in blue and gray, respectively. The number of events was 0 in the concentration of 0.5 pg/μL, suggesting no amplification in this reaction. (Fig. 3). No positive microdroplets could be detected in the reactions using non-GBS strains DNA as templates (data not shown). Therefore, the ddPCR with *CpsE* primer has a satisfactory sensitivity and specificity for GBS detection.

### Repeatability test of ddPCR

**Fig. 4:**
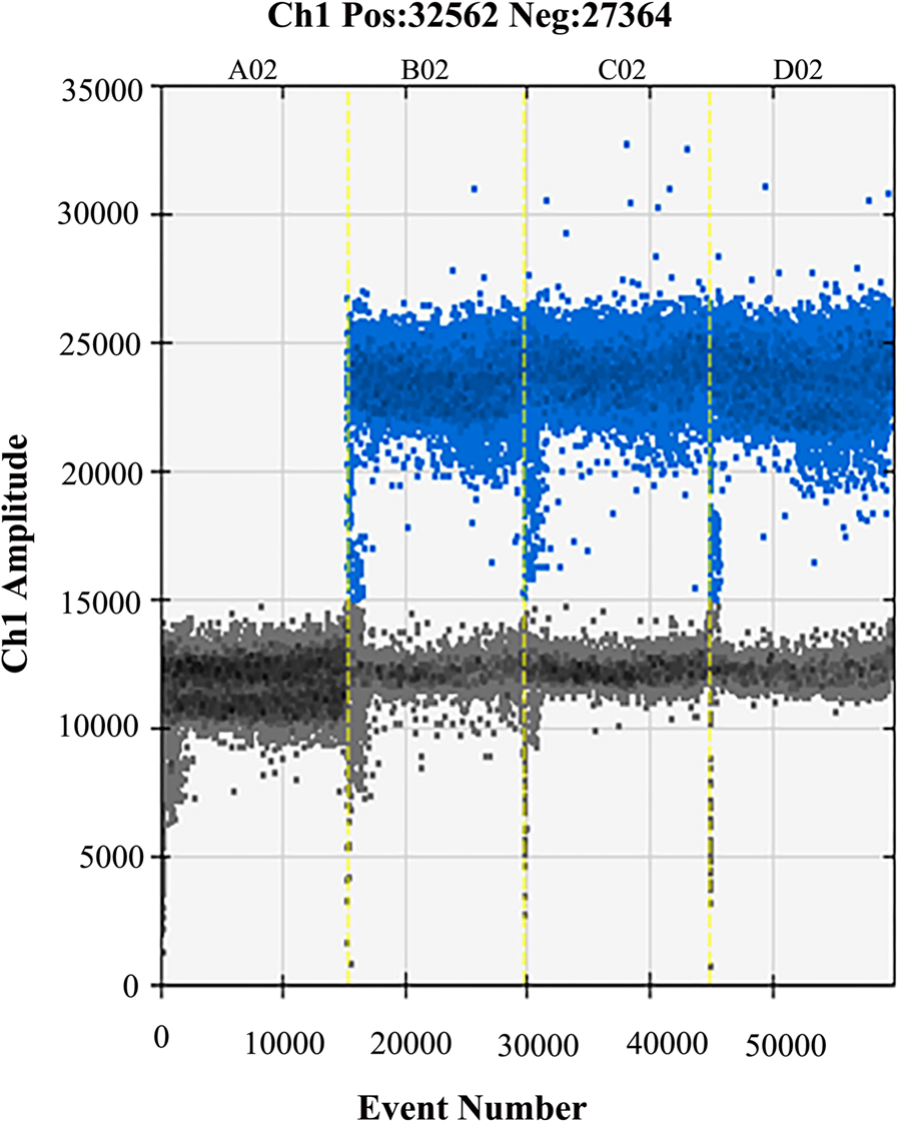
The repeatability test of ddPCR using DNA from the GBS ATCC13813 strain

GBS reference strain was run in triplicate (Fig. 4). The positive events number was 1661, 1560 and 1704, respectively, with a CV of 4.5%, indicating that ddPCR has an excellent repeatability.

## Discussion

*Streptococcus agalactiae* is the leading cause of neonatal pneumonia, infantile septicemia, bacterial meningitis, as well as perinatal infection of pregnant women [15, 16]. Recently, a novel technique, ddPCR, was for DNA quantifications absolutely without depending on the standard curve [17]. Droplet digital PCR is the third generation PCR with higher diagnostic efficiency compared to conventional methods.

Currently, bacterial culture is the gold standard for the identification of GBS infection, but it is time-consuming with limited sensitivity and vulnerable to interference [18]. Also, real-time qPCR requires expensive equipment and only quantifies nucleic acid relatively. In our study, we showed that ddPCR could detect S.agalactiae precisely as low as 5 pg/μL. Moreover, amplification was observed in GBS but not in non-GBS strains, which indicated the high specificity of ddPCR primers. Furthermore, ddPCR had an excellent repeatability with a CV of 4.5%. Given these advantages, it can be used to determine the expression and copy number variation analysis of the target gene [19, 20].

In the present study, the fluorescent dye EvaGreen which binds to dsDNA was used to monitor GBS, but it could cause false-positive results if there existed dimer formations. Thus, the melting curve was analyzed and no double-peak was found, indicating no primer dimer formation. Furthermore, we discovered that the *CpsE* primer amplified the target gene of GBS more effectively than the *Sip* gene.

Some limitations to our study should not be ignored. Firstly, ddPCR is an assay mainly with fluorescent probes. Application with EvaGreen dye may significantly interfere with the experimental results when the primer dimers were forming. Therefore, the demand for primers specificity is very high. Secondly, bubbles in the process will produce less than 12,000 microdroplets. Thus the experiment does not meet the Poisson distribution, leading to inaccurate results. Thirdly, it was reported that the fbs-B gene was targeted with LAMP to identify GBS [5]. Which gene is more effective in detection needs to be further studied. Moreover, we just established a ddPCR method to identify GBS in the present study. However, given the small size of clinical samples that were collected difficultly, further validation should be considered with a larger sample in the next stage of our study. Meanwhile, since the purity of DNA templates are easily affected and varied from different samples, DNA extraction should be optimized to extend the ddPCR testing from the laboratory to further clinical detection.

## Conclusions

In conclusion, our study suggested that ddPCR is a specific, economical and reliable method. However, further validation of larger clinical sample sets are necessary to confirm its value in diagnostic and prove that it is an alternative tool in the clinical detection of GBS.

## Methods

### Bacterial strains

GBS standard strain ATCC13813 was purchased from Shanghai cell bank of the Chinese Academy of Sciences. Eleven non-GBS strains were isolated from the Clinical Laboratory of Third Affiliated Hospital of Guangzhou Medical University and were initially identified by Matrix-Assisted Laser Desorption/Ionization Time of Flight Mass Spectrometry. Non-GBS strains stored at − 70 °C, were used for specificity experiments, including *Candida tropicalis, Candida albicans, Klebsiella pneumoniae, Streptococcus pyogenes, Acinetobacter baumannii, Escherichia coli, Staphylococcus haemolyticus, Candida parapsilosis, Streptococcus anginosus, Enterobacter aerogenes, Pseudomonas aeruginosa.*

### DNA extraction

The ATCC13813 and other non-GBS strains were inoculated onto sheep blood agar and cultured in 37 °C constant thermostatic incubator for 18-24 h. The bacterial colonies picked on the agar plates were inoculated into sterile physiological saline to prepare 1 ml bacterial suspensions [21, 22]. Then the bacterial DNA was extracted and purified according to the instruction manuals of the TIANGEN DNA kit (TIANGEN BIOTECT, Beijing) and was stored at − 20 °C.

### Primer design and synthesis

The sequences of *Streptococcus agalactiae CpsE* and *Sip* gene were obtained from GenBanK [23, 24]. Special primers were designed by Primer Premier 5.0(Premier Laboratories, Canada) and synthesized by Thermo Scientific of Shanghai Trade Co. Ltd. (Table 1). The EvaGreen® dsDNA fluorescent dye (EvaGreen®) was used in this detection.

**Table 1:** Sequences of primers of the Sip and C*psE* gene

Target	Sequences (5′-3′)
*Sip* upstream	*CTGCCAACCACTATGACC*
*Sip* downstream	*CTGCTACAGTTCTTACCG*
*CpsE* upstream	*GCAAAAGAACAGATGGAACAAAGTG*
*CpsE* downstream	*CGCCGTAAGTAGCAACAGAT*

### Droplet digital PCR reaction

The ddPCR reaction was performed in a QX200 Droplet Digital PCR System (Bio-Rad Laboratories, CA) according to the manufacturer’s instruction [11]. Each test was prepared in a 20 μl volume of the reaction mixture, which comprised 10 μL of 2× QX200™ ddPCR™ EvaGreen® Supermix (no dUTP; Bio-Rad), forward and reverse primers and 4 μL of DNA templates. For microdroplets generation, 20ul mixture and 70 ul droplet generation oil were added to the DG8™ cartridge (Bio-Rad), then loaded into a QX200 Droplet Generator (Bio-Rad). Next, microdroplets were transferred into 96-well PCR plate and heat-sealed with foil to prevent aerosol pollution. Then the PCR was performed on a Bulk PCR Thermal Cycler using the following conditions: Pre-denature for 1 cycle at 95 °C for 10 min; denature for 45 cycles at 95 °C for 15 s; anneal and extend for 45 cycles at 60 °C for 1 min. Finally, the fluorescence signal in each plate was analyzed by a QX200 Droplet Reader and QuantaSoft™ Version 1.7.4 [10, 25]. Each reaction adopted negative control and was performed in duplicate.

### Primer screening analysis

Two sets of primers were dissolved into a working solution, and the DNA of the ATCC13813 strain was served as the template. Then the qPCR assay was performed to amplify *CpsE* or *Sip* gene in a SLAN-96P real-time system (HONGSHI. Shanghai). Briefly, we prepared a total volume of 25 μL mixture according to the kit instructions, which contained DNA template, primers, SYBR GREEN dye and PCR Master Mix etc. The reaction procedure was set up identically as the ddPPCR amplification condition mentioned above. Finally, the amplification efficiency was compared to select a better primer for the subsequent assay.

### The sensitivity of ddPCR reaction

DNA obtained from GBS reference strain ATCC13813 was used to determine the limitation of ddPCR assay towards the selected gene. The initial concentration of DNA was adjusted to 5 ng/μL and then diluted four times with sterile saline, i.e. 5000 pg/μL, 500 pg/μL, 50 pg/μL, 5 pg/μL and 0.5 pg/μL. The ddPCR reaction was performed as described above with four concentrations of the DNA template to determine the sensitivity of ddPCR in the GBS test.

### Specificity and repeatability of ddPCR reaction

The DNAs of the GBS ATCC13813 strain and other 11 non-GBS strains were used to assess the specificity of the primers under identical ddPCR conditions. For further repeatability analysis of ddPCR, the reaction was carried out by testing one positive strain and one negative strain three times under identical conditions. Finally, the fluorescence signal was evaluated.

## Data Availability

All data generated or analyzed during this study are included in this published article.

## Abbreviations

GBS: Group B Streptococcus (*Streptococcus agalactiae*)
ddPCR: droplet digital PCR
MALDI-TOF-MS: Matrix-Assisted Laser Desorption/Ionization Time-Of-Flight Mass Spectrometry
LAMP: Loop-mediated isothermal Amplification
CV: Coefficient of Variation
